# Serotonin Transporter Clustering in Blood Lymphocytes of Reeler Mice

**DOI:** 10.1155/2010/396282

**Published:** 2010-04-21

**Authors:** Tania Rivera-Baltanas, Raquel Romay-Tallon, Iria G. Dopeso-Reyes, Héctor J. Caruncho

**Affiliations:** BIOFARMA Research Group, Department of Cell Biology, Faculty of Biology, University of Santiago de Compostela, Santiago de Compostela, 15782 Galicia, Spain

## Abstract

Serotonin transporter clustering is an important feature for regulation of this transporter activity. We used immunocytochemistry to analyze alterations in serotonin transporter clustering in blood lymphocytes of reeler mice. Serotonin transporter immunolabelling is observed mostly as a patchy staining in lymphocytes membranes. Comparison of the number and size of serotonin transporter clusters in wild-type mice, heterozygous reeler mice, and homozygous reeler mice showed an increase in the number and size of clusters in heterozygous reeler mice, but only an increase in clusters size in homozygous reeler mice. Reelin is down-regulated in the brain of schizophrenia, autism, and mood disorders, and is also expressed in blood plasma. There is the possibility therefore that alterations in serotonin transporter clustering in blood lymphocytes associated with a decrease in reelin expression may be operative in some cardiovascular or immune system alterations showing comorbidity with these mental disorders.

## 1. Introduction

Protein clustering into specific membrane domains is known to be of importance for membrane proteins functional regulation: the clustering of neurotransmitter receptors into postsynaptic active sites, the formation of the immunological synapse, the partitioning of membrane proteins into lipid raft domains, and the clustering of membrane proteins to be internalized are good examples of that.

The serotonin transporter (SERT) belongs to the SLC6 family of sodium- and chloride-dependent integral membrane proteins, and is the primary responsible for the recapture of released serotonin from the extracellular space [1, 2]. The clustering of SERT into specific membrane domains such as lipid rafts [3], SERT oligomerization [4, 5], and SERT subcellular distribution [6] appears to be critical for serotonin reuptake activity. SERT is one of the main targets of antidepressant medication, and alterations in SERT expression and activity have been found both in mood and psychotic disorders. In fact, a decrease in SERT binding in blood platelets is one of the best-characterized biomarkers of depression [7], and a similar decrease has also been found in peripheral lymphocytes in depression [8–10]. 

Reelin is a large extracellular matrix protein abundant in brain tissue whose levels are down-regulated in several psychiatric disorders [11–15]. Reelin is also expressed in blood plasma [16], and alterations in reelin plasma levels are also found in different psychiatric disorders such as schizophrenia, mood disorders, and autism [14, 17], although an accurate measurement of reelin plasma levels is not easily accomplished due to its sensitivity to proteolysis and freeze-thawing cycles [18]. 

The primary actions of reelin in the nervous systems are regulating neural migration and synaptogenesis in cortical areas during brain development (i.e., cerebral cortex, hippocampus, olfactory bulb, and cerebellum), and later in stabilizing synaptic contacts onto dendritic spines in the adult brain thereby regulating synaptic plasticity (see [19–21]). These actions are mediated at the molecular level by the interaction of reelin with ApoER2-VLDLR receptors, and bring about the phosphorylation of the adaptor protein DAB1 and activation of nonreceptor tyrosine kinases (see as reviews [20, 21]). In addition, reelin also binds integrin receptors resulting in the upregulation of specific mRNAs translation in dendritic spines [22], and an increase in number and clustering of intramembrane particles (i.e., transmembrane proteins) in postsynaptic membrane domains [23]. 

While reelin actions in the nervous system are well studied, there is not so much knowledge about the possible actions of reelin in blood plasma, although it is known that reelin plasma is mostly secreted by hepatocytes [16], and is processed by plasminogen activator and plasmin [18]. 

Homozygous reeler mice (*R* *e* *l* *n* ^−/−^) show no detectable levels of reelin in plasma, while the happloinssuficient heterozygous reeler mice (*R* *e* *l* *n* ^+/−^) express reelin plasma levels about one half of those in wild-type (*R* *e* *l* *n* ^+/+^) littermates [16], becoming a good model to analyze the effects that reelin down-regulation or null expression may induce in blood cells. 

In the present report, we hypothesize that reelin may have an influence in clustering of membrane proteins in lymphocytes, and analyze the alterations observed in the clustering of SERT in peripheral lymphocytes in *R* *e* *l* *n* ^+/−^ and *R* *e* *l* *n* ^−/−^ mice, in comparison to *R* *e* *l* *n* ^+/+^ mice.

## 2. Materials and Methods

### 2.1. Animals and Collection of Lymphocytes

We have used a total of 48 adult male mice in this experiment: 16 wild-type mice (*R* *e* *l* *n* ^+/+^), 16 heterozygous reeler mice (*R* *e* *l* *n* ^+/−^), and 16 reeler mice (*R* *e* *l* *n* ^−/−^). The animals were obtained from heterozygous breeding pairs (Jackson Laboratory, Bar Harbor, ME). Genotyping was performed by PCR using the following primers: 5′ TAATCTGTCCTCACTCGTCC 3′, 3′ ACAGTTGACATACCTTAATC 5′, 3′ TGCATTAATGTCGACTGTTGT 5′. The PCR products were subsequently analyzed in a gel of 2% agarose. 

Animal handling and maintenance, as well as all experimental procedures, were conducted in accordance with the European Communities Council Directive of 24 November 1986 (86/609/EEC) and Spanish Royal Decree of 14 March 1988 (223/1988/BOE). The performed experiments also received the approval of the ethics committee of the University of Santiago de Compostela. 

Mice were anesthetized by IP injection of 15% chloral hydrate. Blood was collected from the orbits after enucleation of the eyes, followed by euthanasia by cervical dislocation. This procedure allowed to obtain up to 1 mL of blood per animal that was collected with ACD as anticoagulant (85 mM trisodium citrate, 65 mM citric acid, 111 mM anhydrous glucose), at a ratio 1  :  6 (v/v). Blood samples diluted 1  :  1 in 0.9% NaCl were centrifuged in a Percoll gradient for 20 minutes at 800 g, to collect the lymphocytes in the upper layer of the Percoll gradient. The lymphocytes were resuspended in saline solution and centrifuged at 1000 g for 10 minutes. After repeating the procedure, cells were resuspended in 1 mL of saline solution, and fixed for 1 minute in a solution of 1% paraformaldehyde in phosphate buffer at RT. Afterwards, cells were maintained for up to one week at 4°C in 1 mL of saline solution.

### 2.2. Immunocytochemistry

Immunolabelling of SERT was performed by consecutive centrifugation and resuspension of the lymphocyte samples in every step. The procedure involved first the incubation of the samples for 10 minutes at 4°C, in a solution of 100 mL of mice IgG (Sigma), diluted in PBS with 1%BSA, to block membrane immunoglobulins. It followed the incubation for 12 hours at 4°C with a solution of the primary antibody (Rabbit anti SERT, Chemicon) diluted 1  :  100 in PBS with 1%BSA. After washing, the samples were incubated in the dark, for 1 hour at RT with the secondary antibody (Goat antirabbit conjugated with Alexa Fluor 488, Molecular Probes) diluted 1  :  200 in PBS with 1%BSA. After repeated washing, samples were extended on slides and coverslipped and mounted with Moviol medium (Calbiochem). The samples were maintained at −20°C, untill analysis by confocal microscopy. 

Samples were studied and lymphocyte photographs obtained in a laser confocal microscope Leica TCS-SP2. Photomicrographs were collected of 100 lymphocytes per sample. Control experiments by omitting the primary antibody resulted in a lack of immunoreactivity.

### 2.3. Data Analysis and Statistics

SERT labelling as obtained in confocal micrographs was analyzed by using ImageJ 1.42 imaging software (National Institutes of Health). The number of lymphocytes analyzed was 100 per animal (16 animals per group). The imaging system allowed carrying out an automatic counting of the number of SERT clusters per lymphocyte, of the size of those clusters, and also the percentage of the lymphocytes surface occupied by those clusters. In addition, the system allowed performing a surface plot indicating the intensity of fluorescence labeling per cluster. Statistical analysis was done using One way ANOVA followed by Kruskal-Wallis posthoc test. The statistical significance was established at *P* < .05.

## 3. Results

**Figure 1:**
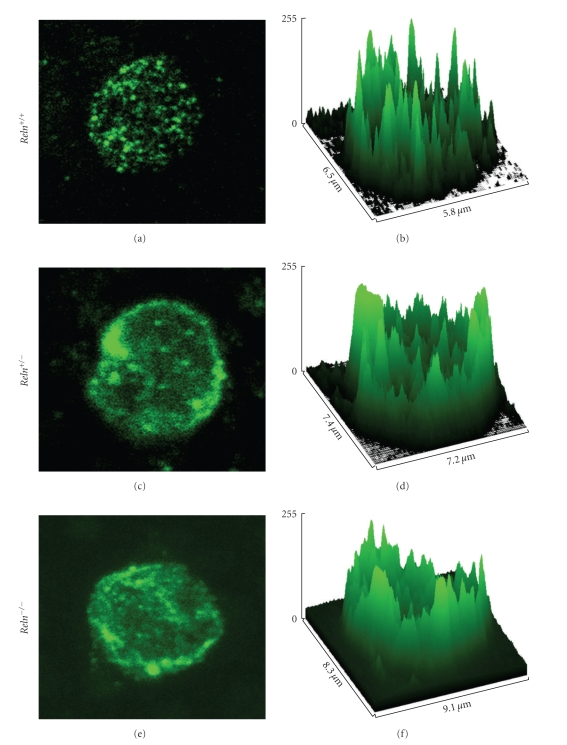
Confocal micrographs (a, c, e) and surface plot graphs (b, d, f) of examples of blood lymphocytes from wild-type (*R* *e* *l* *n* ^+/+^), heterozygous (*R* *e* *l* *n* ^+/−^), and reeler (*R* *e* *l* *n* ^−/−^) mice. Note the increase in SERT-positive clusters size in heterozygous and reeler mice, as well as a decrease in the intensity of labelling as seen in the surface plot analysis.

SERT immunolabelling is mostly evidenced as immunofluorescent clusters observed primarily in the lymphocytes plasma membrane (Figures 1(a), 1(c), and 1(e)). 

The direct observation of SERT immunolabelling in lymphocytes of reeler mice shows that SERT clusters are more clearly seen in *R* *e* *l* *n* ^+/+^ mice lymphocytes, while in both *R* *e* *l* *n* ^+/−^ and *R* *e* *l* *n* ^−/−^ mice lymphocytes, SERT staining is more fuzzy and of a lower intensity, as can be seen in surface plot images (Figures 1(a)–1(f)). 

**Figure 2:**
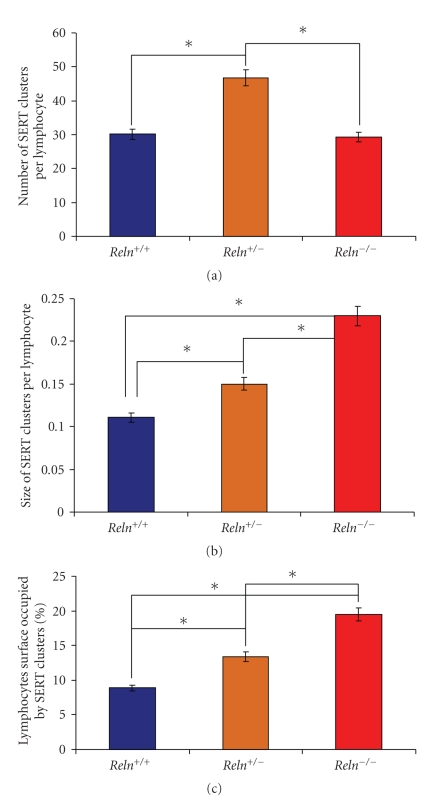
Results of the image analysis of SERT-positive clusters in blood lymphocytes. (a) Number of clusters per lymphocyte. (b) Average size of clusters. (c) Percentage of the lymphocyte surface occupied by SERT-positive clusters.

Statistical analysis of data indicates that the number of SERT immunopositive clusters per lymphocyte is increased about 50% in *R* *e* *l* *n* ^+/−^ mice in comparison with wild-type mice (Figure 2(a) and Table 1), while in *R* *e* *l* *n* ^−/−^ mice the number of SERT immunopositive clusters is similar to that of wild-type mice, and therefore, lower than in *R* *e* *l* *n* ^+/−^ mice (Figure 2(a) and Table 1). 

The average size of SERT immunopositive clusters is increased about 27% in *R* *e* *l* *n* ^+/−^ mice in comparison to wild-type mice, while in reeler mice (*R* *e* *l* *n* ^−/−^), the average size of SERT clusters more than doubles that of wild-type mice (and increase of 109%), and is about 64% larger than in heterozygous reeler mice (*R* *e* *l* *n* ^+/−^ (Figure 2(b) and Table 1). 

The percentage of the lymphocytes surface occupied by SERT immunopositive clusters is also increased in both *R* *e* *l* *n* ^+/−^ mice (a 50% increase) and *R* *e* *l* *n* ^−/−^ mice (an increase of 119%) with respect to wild-type mice (Figure 2(c) and Table 1). In addition, there is an increase of about 47% in the same value in *R* *e* *l* *n* ^−/−^mice in comparison with *R* *e* *l* *n* ^+/−^ mice (Figure 2(c) and Table 1). 

**Figure 3:**
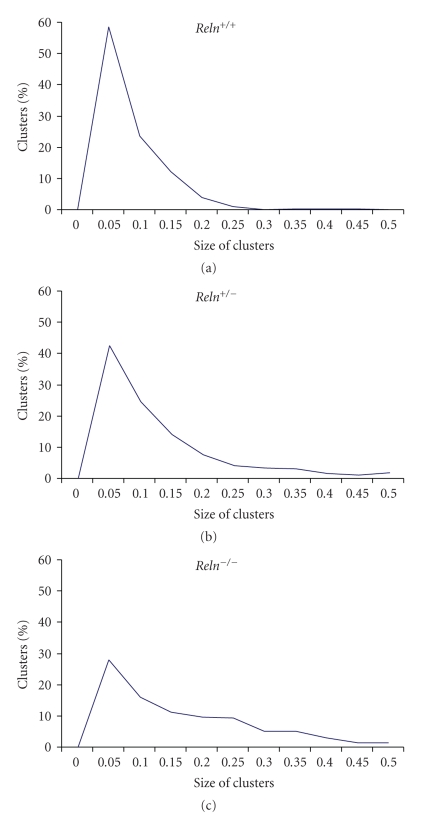
Representation of the distribution of SERT-positive clusters size. Note that the peak of clusters size between 0.05–0.1 *μ*m^2^ is decreased in the heterozygous and reeler mice, while there is a concomitant increase of larger clusters.

The graphic representation of the distribution of SERT clusters size in lymphocytes evidences that about 60% of the clusters are comprised in the interval of 0.05–0.1 *μ*m^2^ in wild-type *R* *e* *l* *n* ^+/+^ mice, while this percentage is only of about 40% in lymphocytes of *R* *e* *l* *n* ^+/−^ mice, and 30% in *R* *e* *l* *n* ^−/−^ mice (Figure 3).

## 4. Discussion

As far as we know this is the first attempt to study alterations of SERT clustering in blood lymphocytes of reeler mice. The results show that a down-regulation of about one-half of plasma reelin levels such as that observed in *R* *e* *l* *n* ^+/−^ mice brings about an increase in the number of SERT clusters and also an increase in clusters size, whereas in *R* *e* *l* *n* ^−/−^ mice (showing null expression of reelin) the number of SERT clusters is similar to that observed in *R* *e* *l* *n* ^+/+^ mice, but of a much larger size than not only those in *R* *e* *l* *n* ^+/+^ mice, but also of those in *R* *e* *l* *n* ^+/−^ mice. 

In case of a possible replication of these studies, it is essential to consider the fixative employed, the time of fixation, and the temperature at which the experiments were performed, because all of these parameters can affect the degree of membrane proteins clustering: for example, experiments of labelling of lipid raft markers showed that an increase in the amount of fixative tends to reduce the labelling of membrane markers, while a decrease in the amount of fixative and/or a decrease in the temperature of fixation tends to increase the size of lipid raft clusters as ascertained by immunolabelling [24]. 

Many reports have focussed on brain and behavioural alterations in both reeler and heterozygous reeler mice (see [25–28]); however, there are no so many studies on blood cells alterations in reeler mice. Interestingly, a report by Green-Johnson et al. [29] shows a suppression of T cell and macrophage function in reeler mice. These authors showed a decrease in proliferation of CD3-positive splenic T cells in *R* *e* *l* *n* ^−/−^ mice (but not in *R* *e* *l* *n* ^+/−^ mice) after activation of the immune system by injection with anti-CD3 antibodies for three days, and also a decrease in production of IL-2, IL-4 and interferon in *R* *e* *l* *n* ^−/−^ mice, while there was only a significant decrease in the production of IL-4 (but not in the other parameters) by *R* *e* *l* *n* ^+/−^ mice [29]. At the time of this publication (1995), there was no evidence of reelin expression in blood plasma, and so the authors were discussing the possibility that the alterations found in T-cell lymphocytes could be related to alterations in the nervous system (mostly in the cerebellum) of *R* *e* *l* *n* ^−/−^ mice. However, with the discovery of reelin expression in blood plasma, it seems more possible that there could be a direct connection between the decrease of reelin levels in blood plasma and the alterations observed in T cells in *R* *e* *l* *n* ^−/−^ mice. Our data shows an increase in SERT clusters size in both *R* *e* *l* *n* ^+/−^ and *R* *e* *l* *n* ^−/−^ mice, although this increase is much higher in *R* *e* *l* *n* ^−/−^ mice where SERT clusters average size is more than double of that in *R* *e* *l* *n* ^+/+^ mice and more than 60% larger than in *R* *e* *l* *n* ^+/−^ mice. In addition, surface plots of SERT immunofluorescence appear to indicate a decrease in the intensity of labeling per cluster that is more pronounced in *R* *e* *l* *n* ^−/−^ lymphocytes, and the number of SERT-positive clusters is increased in *R* *e* *l* *n* ^+/−^ mice but not in *R* *e* *l* *n* ^−/−^ mice. This could be interpreted as an alteration in SERT clustering, reflecting an accumulation of SERT in clusters of small size in *R* *e* *l* *n* ^+/+^ mice, while a decrease in reelin expression brings about the spread out of SERT in clusters of larger size. The clustering of SERT in lipid raft domains is important for SERT functional roles [3], and it is possible that a decrease in reelin levels could bring about an alteration of this clustering. In *R* *e* *l* *n* ^+/−^ mice, the spread out of SERT in lymphocytes membranes is much lower than that in *R* *e* *l* *n* ^−/−^ mice, and in addition there is a 50% increase in the number of SERT clusters in *R* *e* *l* *n* ^+/−^ mice that could represent a compensatory mechanism that is not evident in *R* *e* *l* *n* ^−/−^ mice. Both of these facts could perhaps explain in part the much lower alterations in T cells in *R* *e* *l* *n* ^+/−^ mice than in *R* *e* *l* *n* ^−/−^ mice observed by Green-Johnson et al. [29]. 

Reelin has been shown to induce the expression and clustering of some transmembrane proteins in synaptosomes [23], induces the clustering of its own receptors [30], and regulates NMDA receptor surface trafficking [31], therefore, it is not surprising that there is a spread out of SERT labelling in the membrane of lymphocytes in   *R* *e* *l* *n* ^+/−^ and *R* *e* *l* *n* ^−/−^ mice. In fact, it is possible that reelin may be involved in regulating the clustering of multiple membrane proteins, including some neurotransmitter receptors and transporters. Although brain serotonin levels appear to be unaltered in *R* *e* *l* *n* ^+/−^ mice, both *R* *e* *l* *n* ^+/+^ and *R* *e* *l* *n* ^+/−^ mice show an increase in brain serotonin levels upon early maternal separation of the pups [32]. In addition, at least in *R* *e* *l* *n* ^−/−^ mice there are important alterations in serotonergic innervations in the neocortex [33]. Therefore, we are currently studying possible alterations in SERT clustering in the brain of these mice. Reelin is down-regulated in schizophrenia, autism, and mood disorders, showing a general decrease in different brain areas in schizophrenia, autism, and bipolar disorder, while there could be a more specific decrease in major depressive disorder [11–14]. Reelin levels and its processing are also altered in blood plasma of schizophrenia, autism, and mood disorders [14, 17]. Interestingly, brain and plasma reelin levels are highly downregulated in autism [14, 34–38], a disorder that is also characterized by blood hyperserotonemia [39, 40]. Taking into account the alterations in SERT clustering in lymphocytes of reelin-deficient mice shown in this report, one should consider the possibility of a similar alteration in lymphocytes of autistic patients that have both low plasma reelin levels and high plasma serotonin levels. In addition, it would be of interest to study a possible relation between the low levels of plasma reelin and the blood hyperserotonemia observed in these patients, as well as a possible increase in serotonin levels in blood plasma in reelin-deficient mice. 

The results of the present report also invite to consider the actions of plasma reelin actions in SERT clustering in lymphocytes as a possible factor of importance in understanding the comorbidities between some immune and/or vascular systems alterations and these mental disorders. 

## Figures and Tables

**Table 1:** Statistical analysis of SERT-positive clusters in blood lymphocytes of *R* *e* *l* *n* ^+/+^, *R* *e* *l* *n* ^+/−^, and *R* *e* *l* *n* ^−/−^ mice.

SERT clusters	*R* *e* *l* *n* ^+/+^	*R* *e* *l* *n* ^+/−^	*R* *e* *l* *n* ^−/−^
Number	30.8 ± 1.08	46.7 ± 1.47^a^	29.3 ± 0.72
Size	0.11 ± 0.003	0.14 ± 0.003^b^	0.23 ± 0.004^c^
% of lymph. surface	8.88 ± 0.32	13.3 ± 0.22^d^	19.5 ± 0.59^e^

Statistical significance set at *P* < .05.

a, b, d. Different than in *R* *e* *l* *n* ^+/+^ and *R* *e* *l* *n* ^−/−^ mice.

c, e. Different than in *R* *e* *l* *n* ^+/+^ and *R* *e* *l* *n* ^+/−^ mice.
